# Carbon monoxide breath test assessment of mild hemolysis in Gilbert's syndrome

**DOI:** 10.1097/MD.0000000000019109

**Published:** 2020-02-14

**Authors:** Ling-Ling Kang, Yong-Jian Ma, Hou-De Zhang

**Affiliations:** a Department of Gastroenterology, Nanshan Hospital, Guangdong Medical University; b Guangdong Breath Test Engineering and Technology Research Center; c Institute of Breath Test Research, Shenzhen University, Shenzhen, China.

**Keywords:** erythrocyte lifespan, Levitt's CO breath test, red blood cell, UGT1A1, unconjugated hyperbilirubinemia

## Abstract

**Background:**

Mild hemolysis is difficult to determinate by traditional methods, and its role in Gilbert's syndrome (GS) is unclear. The main aims were to inspect the erythrocyte (RBC) survival in GS by using Levitt's carbon monoxide (CO) breath test and to assess its contribution to unconjugated hyperbilirubinemia.

**Methods:**

Fifty subjects with GS and 1 with type-II Crigler–Najjar syndrome (CN2) received RBC lifespan measurement with Levitt's CO breath test. Mean RBC lifespan was compared with normal referral value. Correlations of serum total bilirubin (TB) with RBC lifespan, blood panel data, demographic factors, and uridine diphosphate glucuronosyltransferase (*UGT1A1*) mutation load were calculated by Spearman analysis. Susceptibility factors for mild hemolysis were analyzed by multivariate regression analysis.

**Results:**

The mean RBC lifespan of the GS subjects was significantly shorter than the normal reference value (95.4 ± 28.9 days vs 126 days; *t* = −7.504, *P* < .01), with 30.0% below the lower limit of the normal reference range (75 days). The RBC lifespan of the participant with CN2 was 82 days. Serum TB correlated positively with *UGT1A1* mutation load (*γ* = 0.281, *P* = .048), hemoglobin (*γ* = .359, *P* = .010) and hematocrit (*γ* = 0.365, *P* = .010), but negatively with RBC lifespan (*γ* = −0.336, *P* = .017). No significant susceptibility factors for mild hemolysis were found.

**Conclusions:**

The results indicate that mild hemolysis indeed, exists in a portion of patients with GS and might serve as an important contributor to unconjugated hyperbilirubinemia in addition to *UGT1A1* polymorphism. Further studies on the mechanism and the potential risks in various medical treatments might be wanted.

## 1 Introduction

Gilbert's syndrome (GS) is a well-known phenotypically heterogenous hereditary condition characterized by mild, chronic unconjugated hyperbilirubinemia in the absence of liver disease or overt hemolysis.^[1–3]^ The pathogenesis of GS has been linked to a congenital mutation in the gene encoding uridine diphosphate glucuronosyltransferase (*UGT1A1*) that reduces synthesis of *UGT1A1*, which functions to conjugate bilirubin in hepatocytes.^[1,4,5]^ GS is generally non-symptomatic and the mild elevated blood unconjugated bilirubin may even have the benefits of increasing antioxidation potential and reducing the risk of type 2 diabetes, cardiovascular disease, and certain cancers.^[6–12]^ In recent years; however, GS has been associated with increased risk for gallstones.^[13–20]^ What is more, because *UGT1A1* plays a role in drug metabolism, its hypo-expression in GS may reduce the efficacy of and increase the toxicity of some chemotherapeutic drugs like irinotecan used to treat leukemia, colorectal cancer, lung cancer, and ovarian cancer.^[21–28]^

Although unconjugated hyperbilirubinemia in persons with GS is considered to be of nonhemolytic origin, early reports described mild hemolysis in 30% to 80% of persons with GS based on slightly shortened erythrocyte (RBC) lifespan, relative to normal values, estimated by ^51^Cr-labeled RBC re-transfusion tests.^[29–33]^ However, these studies had important limitations, namely small sample sizes and RBC lifespan estimations based on only ^51^Cr-labeled RBC half-life data rather than whole RBC lifespan data. The main reason RBC lifespan is estimated in this way is that measuring the whole RBC lifespan with ^51^Cr-labeling techniques requires multiple venesections over a series of time points that can extend into several months. Thus, although ^51^Cr-labeled RBC re-transfusion tests are reliable for hemolysis diagnosis when performed to completion, they are cumbersome and time-consuming. Because of these technical drawbacks, the mechanism of mild hemolysis in GS, its role in GS pathogenesis, its contributions to unconjugated hyperbilirubinemia, and its effect on drug safety have remained unclear.

Based on the knowledge that endogenous carbon monoxide (CO) originates mainly from degraded RBC, a team lead by Levitt's developed a simple, rapid, and accurate CO breath test to determinate RBC lifespan.^[34,35]^ Hence, the aims of this study were to assess the mild hemolysis in GS by the use of Levitt's CO breath test, and to evaluate its contribution to the unconjugated hyperbilirubinemia.

## 2 Methods

### 2.1 Subjects

The study participants included 50 patients with GS (36 males and 14 females; median age of 29.0 years, range 12.0–63.0 years) and 1 30-year-old female patient with type-II Crigler–Najjar syndrome (CN2). CN2, a rare hereditary moderate to severe unconjugated hyperbilirubinemia, is caused by the same UGT1A1 gene mutation as GS. All 51 participants met the applicability requirements for CO breath testing (>7 years old, nonsmoker).^[36]^ GS diagnosis criteria were adapted from those of Bosma et al^[1]^ with some modification. Briefly, we applied the following diagnostic criteria: >6 months history of serum TB elevation in the range of 20 to 90 μmol/L (1.2–5.3 mg/dL) as determined by the Diazo reaction, with unconjugated bilirubin ≥99% in a high-performance liquid chromatography (HPLC) assay; genetic mutation test of UGT1A1 was positive; normal liver function test results (ie, serum albumin level, globulin level, enzymatic indexes of liver damage, and enzymatic cholestasis indexes). Overt hemolysis was excluded on the basis of hemoglobin (Hb) above normal lower limit, normal reticulocyte (RET) counts, and a normal microscopy of blood smear.

The patient with CN2 was diagnosed initially in another hospital, where she had presented with long-term juvenile-onset jaundice. She had a healthy jaundice-free daughter. Her serum TB varied in the range of 185.2 to 200 μmmol/L, with <15% directed bilirubin. Her jaundice and elevated serum TB were resolved with oral phenobarbital, but would relapse upon phenobarbital withdrawal. Histology of a liver biopsy sample was generally normal but with lipofuscin deposition. A homozygotic *UGT1A1* dual mutation (p.[G71R;Y486D]) was confirmed by next generation sequencing; her daughter was likewise confirmed to be a heterozygous carrier of this dual-mutation allele.

The study protocols were approved by the Review Boards of Nanshan Hospital, Guangdong Medical University. The experiments were carried out in accordance with the Declaration of Helsinki. All participants provided written informed consent upon being enrolled in the study.

### 2.2 RBC lifespan measurement

Levitt's CO breath test was conducted with an ELS TESTER automated machine (Seekya Biotec Co. Ltd, Shenzhen, China); Peripheral venous blood samples for blood panel tests were collected from each participant on the same day as breath sampling. The principle of Levitt's CO breath test is that endogenous CO in the breath originates mainly (∼70%) from heme oxidation during Hb degradation following RBC rupture, such that the total capacity of CO from Hb divided by the CO quantity released per day equates to mean RBC lifespan.^[34,35,37–39]^ The test was carried out according to the instrument manufacturer's instructions. Briefly, breath samples were collected in the morning without a fasting requirement; each subject was instructed to take a deep inspiration, hold his or her breath for 10 seconds, and then exhale into a collection system that discards the first 300 mL of transferred air (dead space) and then directs subsequent alveolar air into a foil bag (volume, 1500 mL). If needed, the procedure was repeated until the collected air sample reached the collection bag capacity. The filled bag was detached and sealed. Atmospheric samples were collected just after breath sampling. Alveolar air and atmospheric samples were stored at room temperature and analyzed within 2 days of collection. The alveolar and environmental foil air sample bags were connected to inlet ports and Hb data obtained from the aforementioned blood testing were inputted; then, automatic measurement by the ELS TESTER was initiated by pushing the start button on the machine. The instrument determines alveolar endogenous CO concentration (endoPco) by nondispersive infrared spectroscopy of the paired alveolar and environmental air samples and then calculates RBC lifespan according to the simplified Levitt's formula: RBC lifespan (days) = 1380 × [Hb]/endoPco. The RBC lifespan result is reported 15 minutes after initiation of analysis with the start button. The mean normal RBC lifespan reference value of Levitt's CO breath test was 126 days, with a range 75 to 177 days, which was similar to that obtained by the golden classical standard ^51^Cr, ^15^N-glycine, and biotin labeling techniques (mean 120 days, range 70–140 days).^[36]^

### 2.3 Genetic analysis and blood tests

All laboratory tests, except genetic sequencing and HPLC to determine TB, were performed in the Clinical Laboratory of Nanshan Hospital. Sanger sequencing of *UGT1A1* was carried out by the Beijing Genomics Institute using genomic DNA extracted from leucocyte (WBC) isolated from 1 mL of peripheral venous blood; all 5 exons and the *UGT1A1-*adjacent phenobarbital responsive enhancer module were amplified by polymerase chain reaction, the purified products were subjected to Sanger sequencing in an ABI 3730XL genetic analyzer (Applied Biosystems, Foster City, CA), and the sequencing data obtained were analyzed in Sequencher software at the Beijing Genomics Institute. Detected genetic variants were confirmed by bidirectional direct sequencing with a second independent polymerase chain reaction fragment.

HPLC to determine serum TB was conducted with a Dionex Ultimate 3000 chromatograph ((Thermofisher Scientific, USA)) at the Institute of Breath Test Research, Shenzhen University as described by Singh and Bowers.^[40]^ Samples were prepared by dilution in ascorbic acid/dimethyl sulfoxide and filtration to remove solid materials. Diluted serum was injected directly into a 150 mm × 4.6 mm I.D. HPLC column packed with 5-μm Supersil C4 silica and 300-A pores (Elite Analytical Instruments Co., Ltd., Dalian, China). Bilirubin species were eluted with a water-isopropanol gradient. HPLC enables fractionation and quantitation of 4 bilirubin species: α (unconjugated bilirubin), β (bilirubin monoglucuronide), γ (bilirubin diglucuronide), and δ (bilialbumin). The α elution peak is highly predominant in normal healthy subjects as well as in patients with GS or CN2.

### 2.4 Statistics

Normally distributed data are reported as means ± standard deviations. Nonparametric data are reported as medians with inter-quartile ranges. Enumeration data are expressed as percentages. Student *t* test and Wilcoxon rank-sum or Chi-square tests were applied to analyze continuous variables and categorical variables, respectively. Spearman analysis was used to analyze correlations between TB and target variables. Multivariate linear regression analysis was used to assess factors that may affect RBC lifespan. Univariate analysis of variables with a *P* ≤ .10 and variables of clinical value were included in the multivariate linear regression analysis (significance criterion, *P* < .05). The resultant odd ratios are reported with 95% confidence intervals. All data analyses were conducted in SPSS 22.0 for Windows (SPSS, Chicago, IL).

## 3 Results

### 3.1 Baseline characteristics

All 51 patients completed the study. The median serum TB value obtained for the 50 patients with GS was 40.5 (30.1–48.0) μmol/L. Their *UGT1A1* genotypes were as follows: UGT1A1∗28 in 39 cases (78%); UGT1A1∗60 in 35 cases (70%); UGT1A1∗6 in 26 cases (52.0%); UGT1A1∗27 in 5 cases (10.0%), and UGT1A1∗7 in 1 case (2.0%). Regarding the number of *UGT1A1* mutations (*UGT1A1* mutation load) present, 6 patients (12.0%) had a single mutation, 17 patients (34.0%) had 2 mutations, and 27 patients (54.0%) had 3 or more mutation sites. Their routine blood test results (samples taken on the day of the breath test) were as follows: RBC, 5.1 (4.7–5.3) × 10^12^/L; Hb, 152.0 ± 15.0 g/L; hematocrit (Hct), 45.3 (42.5–47.8)%; Ret 1.4 ± 0.4%, WBC 5.5 (4.9–6.6)× 10^9^/L; and platelet, 212.0 (198.5–259.0)× 10^9^/L. We found that 8 patients (16.0%, all males; Table 1) had a Hb higher than the diagnostic threshold for erythrocytosis (≥165 g/L for men, ≥160 g/L for women).^[41]^ The 30-year-old female patient with CN2 had a serum TB concentration of 186.2 μmmol/L, Hb of 153 g/L, and Ret of 3.44%. Her *UGT1A1* genotype was p.[G71R;Y486D] homozygous.

**Table 1:**
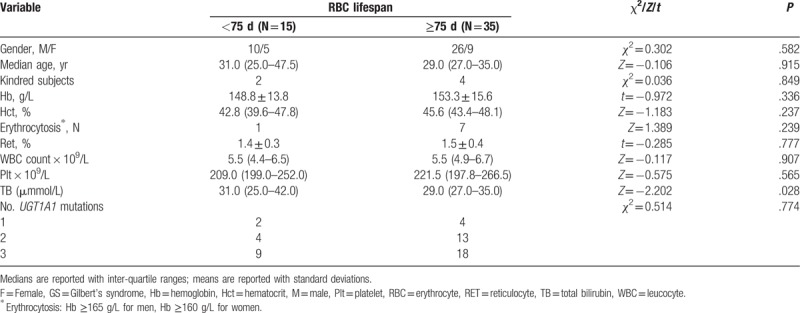
Comparison of demographics and laboratory results of patients with GS across RBC lifespan groups.

### 3.2 RBC lifespan

The mean RBC lifespan for the 50 patients with GS was 95.4 ± 28.9 days (range, 44–183 days), which is significantly shorter than the average normal value of 126 days (*t* = −7.504, *P* < .01). Among them, there were 15 patients (30.0%) who had RBC lifespans below the normal reference lower limit of 75 days, consistent with mild hemolysis. The RBC lifespan of the only participant with CN2 was 82 days, which is within the normal range.

### 3.3 Factors related to unconjugated hyperbilirubinemia

**Figure 1:**
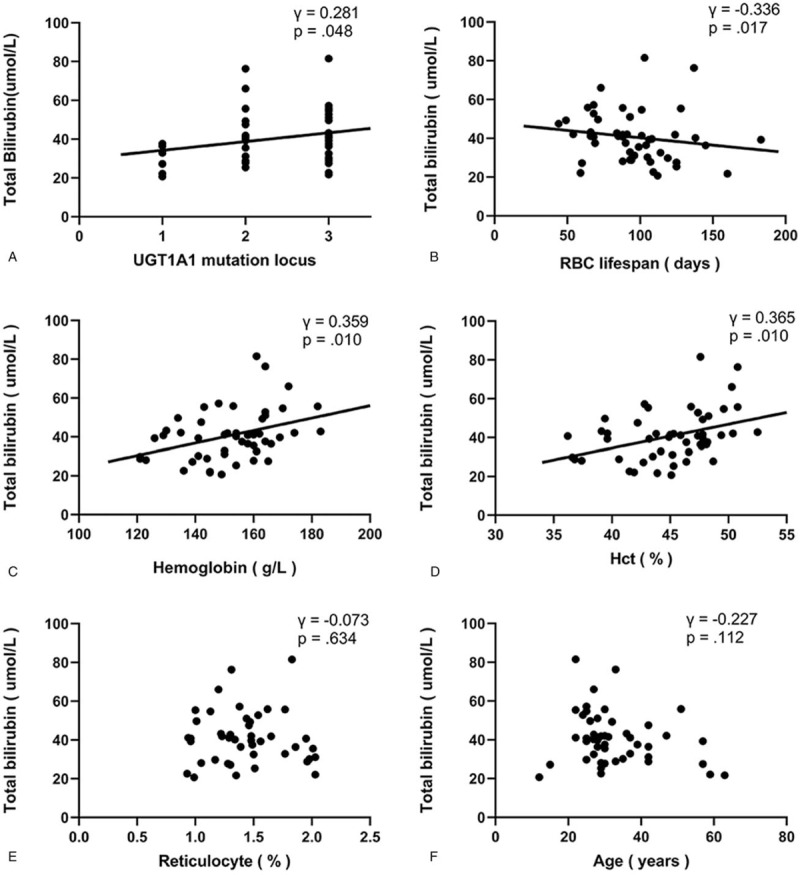
Plots of TB correlations with variables of interest. Relationships of serum TB with RBC lifespan determined by Levitt's CO breath test (A), *UGT1A1* mutation load (B), Hb (C), Hct (D), Ret (E), and age (F) are shown. CO = carbon monoxide, Hb = hemoglobin, Hct = hematocrit, RBC = erythrocyte, RET = reticulocyte, TB = total bilirubin.

Wilcoxon rank-sum testing indicated that the median serum TB values for men with GS (41.1 μmol/L) and women with GS (34.8 μmol/L) were statistically similar (*z* = −1.448, *P* = .148). Spearman bivariate analysis of TB correlations with *UGT1A1* mutation load, RBC lifespan, Hb, Hct, Ret, and age are shown in Figure 1. Briefly, TB correlated positively with *UGT1A1* mutation load (*γ* = 0.281, *P* = .048), Hb (*γ* = 0.359, *P* = .010), and Hct (*γ* = 0.365, *P* = .010), correlated negatively with RBC lifespan (*γ* = −0.336, *P* = .017), and did not correlate significantly with Ret (*γ* = −0.073, *P* = .634) or age (*γ* = −0.227, *P* = .112).

### 3.4 Susceptibility factors for mild hemolysis

Comparison of blood panel and demographic variables between patients with RBC lifespans <75 days and those with RBC lifespans <75 days revealed significant differences in sex distribution, Hb, and TB between these RBC lifespan groups (Table 1). Subsequent multivariate linear regression analysis showed that none of the variables was a significant susceptibility factor for the shortening of RBC lifespan.

## 4 Discussion

In this study, we measured RBC lifespan in 50 patients with GS and 1 patient with CN2 using a simple and rapid automated Levitt's CO breath test method. To our knowledge, our cohort involved the largest GS sample to be subjected to RBC lifespan measurement in a single study. We found that the mean RBC lifespan of our GS patient group was significantly lower than the normal reference mean value and that 30.0% of the patients had a RBC lifespan below the lower limit of the normal reference range. These results confirmed that mild hemolysis was present in almost a third of patients with GS.^[29–33]^

Serum TB is highly variant across GS patients and fluctuates within individuals across different time periods.^[42]^ Although *UGT1A1* polymorphism is associated with the pathogenesis of GS, *UGT1A1* genotype does not fully explain the variability among patients nor the variability over time within patients.^[1]^ Here, we observed only a weak positive correlation between *UGT1A1* mutation load and bilirubin level (*γ* = 0.281), which suggests that there are other factors that contribute to GS unconjugated hyperbilirubinemia. Indeed, levels of serum unconjugated bilirubin are determined by a series of processes, including bilirubin production, bilirubin uptake, and hepatocyte membrane-binding of bilirubin.^[42]^

Epidemiologically, the incidence of GS is higher in males than in females, and hyperbilirubinemia usually appears either in the neonatal period or after puberty.^[43]^ Meanwhile, undernourishment and drugs like Rifamycin exacerbate hyperbilirubinemia in persons with GS.^[44]^ Other factors that might influence hyperbilirubinemia include body mass index, RBC mass, and hemolysis.^[3,42,45,46]^ The present data do not provide evidence of a gender or age effect on bilirubin level in patients who have developed hyperbilirubinemia. Unfortunately, body mass index data were not recorded in this study. Meanwhile our findings of TB correlations with Hb and Hct are consistent with prior reports.^[45,46]^ With respect to the potential relationship between mild hemolysis and bilirubin levels, prior reports have been largely speculative with little statistical confirmation.^[29–33]^ Our finding of a negative correlation between RBC lifespan and serum TB suggests strongly that RBC turnover rate is an important factor affecting serum bilirubin levels in patients with GS.

Clarification of the mechanism underlying the development of mild hemolysis in GS is beyond the scope this study and few previous studies had been reported data that elucidate this issue. Although our univariate analysis showed that serum TB was significantly higher in persons diagnosed with GS with a shortened RBC lifespan than in those with an RBC lifespan within the normal range, subsequent multivariate linear regression did not reveal any significant susceptibility factors.

The cytotoxicity of high unconjugated bilirubin levels is well known in neonatal kemicterus and in vitro research.^[7]^ Our Spearman analysis showed a negative correlation between TB and RBC lifespan, such that participants with higher serum TB tended to have shorter RBC lifespans. This correlation is consistent with the possibility that mild hemolysis might be a sequela of unconjugated hyperbilirubinemia. However, our CN2 participant had a normal RBC lifespan despite a very high unconjugated bilirubin value. Similarly, in 1971, Bloomer et al^[47]^ described the case of 7-year-old child with CN2 with a high serum TB (24.0 mg/100 mL; 410.4 μmol/L), but low conjugated bilirubin (0.34 mg/100 mL; 5.8 μmol/L), who had an RBC half-life within normal range (29.1 days) according to ^51^Cr-labeled RBC analysis. The disassociation between TB and RBC lifespan in subjects with CN2 argues against the notion that mild hemolysis in GS patients may be secondary to unconjugated hyperbilirubinemia.

For treatment planning purposes, it is important to know whether GS patients with mild hemolysis are at a heightened risk of drug-induced hemolysis or other side effects.^[23]^ Levitt's CO breath test used in this study represents a highly convenient tool that can be used to screen and monitor patients for the development of hemolysis before prescribing medications that may be contraindicated in patients with hemolysis and during ongoing pharmacotherapy interventions, respectively.

The main limitation of this study is that the Levitt's CO breath test method does not reveal whether hemolysis is the result of excessive RBC destruction, ineffective hematopoiesis, or both. That is, our measurements did not distinguish between the components that underlie RBC lifespan.^[36,37,48,49]^ Notwithstanding, the method does enable the presence of mild hemolysis in GS patients to be identified with ease.

In conclusion, the simple and rapid Levitt's CO breath test was used to measure the RBC lifespan of patients with GS in this study. The research confirmed that some patients were accompanied with mild hemolysis, which was an important contributors to the unconjugated hyperbilirubinemia of GS. Further studies on the mechanism and the potential risks in various medical treatments might be wanted.

## Acknowledgments

The authors thank Drs. Jian-Xue Hu, Yun Ling, Rong-Ping Yang, Xue-Feng Mu, Wei Ding, Xu Liao, and Ze-Lin Liu for their enthusiastic help with subject enrollment, and thank Mr. Jun-Feng Luo for his statistical advice.

## Author contributions

**Writing – original draft:** Ling-Ling Kang, Yong-Jian Ma, Houde Zhang.

Houde Zhang orcid: 0000-0001-8680-0073.
